# Editorial: Food systems for nutrition: converging economic, social, and environmental sustainability

**DOI:** 10.3389/fnut.2026.1896421

**Published:** 2026-07-09

**Authors:** Shalander Kumar, Abhishek Das, Shibani Ghosh, Victor Afari-Sefa, Jörn Oliver Schmidt

**Affiliations:** 1 Transforming Agrifood System, International Crops Research Institute for the Semi-Arid Tropics, Patancheru, India; 2 College of Agriculture and Life Sciences, Cornell University, Ithaca, NY, United States; 3 Transforming Agrifood System, International Crops Research Institute for the Semi-Arid Tropics, Kano, Nigeria; 4 WorldFish, Penang, Malaysia

**Keywords:** economic sustainability, environmental sustainability, food security, food systems for nutrition, social sustainability

The global food system stands at a critical juncture. Feeding a world population projected to reach nearly 10 billion by 2050 demands not just more food but better food, including production, distribution, and consumption that are ecologically sound, economically viable, and socially just. The urgency of this challenge is underpinned by a set of converging crises that are both independently severe and deeply interconnected. Roughly 733 million people still face hunger, while over three billion cannot afford a healthy diet (1). These challenges are unevenly distributed, with coastal and island communities dependent on aquatic food systems often overlooked by terrestrial-focused interventions. Food systems account for approximately 34% of global greenhouse gas emissions (2). Diet-related non-communicable diseases have emerged as the leading cause of preventable premature mortality worldwide (3). Climate change is intensifying risks to food production in precisely the regions that are already most food-insecure. These are not separate emergencies to be addressed by separate disciplines. They are symptoms of a food system that prioritizes short-term productivity and economic efficiency over long-term human and planetary health, leaving vulnerable communities exposed to shocks they neither created nor can easily absorb. Addressing these symptoms demands approaches that simultaneously advance economic sustainability, social inclusion, and environmental stewardship, the triple convergence that frames this Research Topic.

The concept of food system transformation has gained traction in international policy discourse, most visibly through the 2021 UN Food Systems Summit and the Sustainable Development Goals framework. Yet rhetoric has frequently outpaced practice. The gap between ambitious global commitments and ground-level realities, whether on smallholder farms in Sub-Saharan Africa, in hospital kitchens in the Middle East, or in publicly funded nutrition programs in the United States, remains vast and, in many contexts, widening. Closing this gap requires not only better science but also better translation of science into policy and practice, including understanding why transformations succeed or fail in real-world contexts.

This Research Topic, published across *Frontiers in Nutrition* and *Frontiers in Sustainable Food Systems*, was designed to advance precisely this transition. It invited interdisciplinary contributions that could not separate agricultural productivity from nutritional outcomes, economic incentives from social equity, or environmental sustainability from cultural acceptability. The response was both wide and substantive: 23 original articles from researchers and practitioners spanning six continents, employing methods as varied as dietary optimization modeling, multi-year household panel data analysis, participatory trait-preference surveys, laboratory-to-practice aquaculture engineering, qualitative clinic-staff interviews, and 50-year historical analyses of agricultural production trends. Together, they constitute a genuinely integrated and internationally grounded contribution to the science and practice of sustainable food systems.

The breadth of this Research Topic is one of its strengths, but it warrants some navigational guidance. We have organized our discussion of the 23 contributions into four thematic groupings that reflect the major intellectual threads running through the Research Topic. The first addresses the transformation of agricultural production itself: agroecological and regenerative approaches, the behavioral dimensions of farmer adoption, and innovative circular production systems. The second examines sustainable diets and food environments: dietary optimization and its trade-offs, the distributional costs of dietary transition, the environmental footprint of nutritional guidelines, and the structural and behavioral factors that shape what people actually eat. The third focuses on food loss and waste, both the quantified scale of institutional waste and the relational dynamics of waste reduction among food professionals. The fourth addresses governance, equity, and inclusive food policy across diverse contexts from the Pacific Island governance structures to medically tailored meal programs in the United States. These groupings are not watertight compartments; cross-cutting themes of gender equity, climate resilience, and economic accessibility run through all four. What they offer is orientation rather than partition.

## 1 Transforming agricultural production: agroecology, farmer behavior, and innovative production systems

Agriculture is where food systems begin and where many of their most consequential trade-offs are established. The seven contributions in this grouping examine how production practices, farmer motivations, and system designs can be reoriented toward outcomes that are simultaneously more nutritious and more ecologically sound, drawing on evidence from India, China, Malawi, West Africa, including one from China's aquaculture sector.

Divyanshu et al. open the section with a field-level comparison of nature-friendly and conventional apple cultivation in Himachal Pradesh, India. Farms that had transitioned away from chemical-intensive inputs demonstrated healthier soil organic matter profiles, reduced disease incidence, and greater agro-biodiversity. The study is candid about transitional costs, yields, and revenues, and acknowledges that they face short-term pressure, but provides grounded evidence that ecological gains are achievable and that market linkages for premium, nature-friendly produce are a necessary complement to agronomic change. Rosier et al. broaden this perspective considerably in their review of regenerative agriculture, making the case that restoring soil health is a nutritional as much as an environmental imperative. Their evidence linking soil microbiome diversity to crop micronutrient density argues that the documented decline in nutrient density of many staple crops over recent decades reflects, in part, the depletion of soil biological communities under industrial farming. Their call for a unified but flexible certification framework for regenerative agriculture reflects the difficulty of scaling inherently context-specific practices without losing their ecological integrity.

The behavioral dimensions of agricultural transition receive rigorous attention from two complementary Chinese studies. Zhang, Mao et al. examine how livelihood capital, including physical, human, financial, social, and natural, shapes green production behaviors among professional grain farmers in Jiangxi Province, while Zhang, Cai et al. apply an integrated Technology Acceptance Model and Theory of Planned Behavior framework to understand the psychological and normative drivers of farmers' willingness to cultivate high-quality rice. Together, they make the point that policy too often ignores: sustainable practice adoption is mediated by multiple forms of capital and subjective perceptions of ease, usefulness, and social expectations. Interventions attending only to the technical or economic dimensions of adoption are likely to disappoint.

Munthali et al. contribute field evidence from Malawi, where conservation agriculture technologies, including crop rotation, intercropping, mulching, and no-till, have generated meaningful gains in food production and food security in Mzimba district, while candidly mapping the informational and economic barriers that continue to slow uptake. Mitchodigni et al. add a critical market lens, mapping variety trait preferences for three traditional African vegetables, including amaranth, jute mallow, and okra, among farmers, traders, and consumers in Benin and Mali. Their finding that market acceptability criteria are at least as important as agronomic traits to value chain actors carries direct implications for plant breeding programs that have historically prioritized yield and micronutrient content above all else. Sustainable agriculture must produce what markets and households will actually value, buy, and eat. Finally, Yang and Lu present a laboratory-to-practice case study of endogenous algal consortia systems that treat nutrient-laden aquaculture wastewater in China while generating biomass recoverable for feed and fertilizer, a vivid embodiment of the circular economy logic the food system urgently needs, and a reminder that aquatic production systems offer distinctive pathways for resource efficiency and nutritional output that are too rarely integrated into mainstream food systems thinking.

## 2 Sustainable diets, food environments, and the challenges of dietary transition

What people eat is shaped by what is available, affordable, and desirable, and those three determinants rarely converge when sustainability is the goal. The eight contributions here examine dietary quality and change from environmental, economic, behavioral, and gender perspectives, spanning New Zealand, the Netherlands, Switzerland, Brazil, Bangladesh, Uruguay, and Turkey, and collectively illuminating why dietary transition is so difficult and where levers might most productively be applied.

Tavan et al. use the iOTA dietary optimization model to surface a finding as important as it is uncomfortable: the diets with the lowest greenhouse gas emissions or the lowest monetary cost are precisely those populations find least acceptable, failing on palatability, cultural familiarity, and culinary tradition. This acceptability-sustainability gap is the central implementation challenge for dietary transition policy, and the paper argues persuasively that acceptability must be a binding constraint in optimization models rather than an afterthought. Vellinga et al. extend the cost dimension to the Dutch context, confirming that diets optimized for both health and environmental sustainability tend to cost more and that this burden falls disproportionately on lower-income households, a distributional finding that too often disappears in debates focused on aggregate environmental impact. Bashiri et al. respond with a methodological contribution, proposing multi-objective optimization frameworks linked to the Behavior Change Wheel to help policymakers identify intervention combinations that are simultaneously nutritionally adequate, environmentally responsible, economically accessible, and behaviorally feasible.

Crosnier et al. assess the carbon, water, and land footprints of Swiss national dietary guidelines under both conventional and organic production systems, finding that organic agriculture does not automatically improve environmental performance across all dietary configurations. The environmental footprint of a diet is jointly determined by what is eaten and how it is produced, a nuanced conclusion that cautions against single-lever policy approaches. Miele et al. bring a striking 50-year perspective from Brazil, tracing how a massive structural shift to soy and commodity crop monocultures has generated serious environmental risks while paradoxically intersecting with worsening nutritional outcomes for women, particularly during pregnancy. Food system transformation at scale generates distributional and health consequences that require anticipatory governance.

Mastura et al. provide panel data evidence from Bangladesh that rice price volatility significantly reduces dietary diversity in farm households, with women and children bearing disproportionate nutritional costs, a clear signal that commodity price stabilization and social protection mechanisms belong in the nutrition policy toolkit. On the behavior side, Machín et al. use the Social Ecological Model to explore barriers to fruit and vegetable consumption among Uruguayan adults, finding that nutrition knowledge alone is far from sufficient and that economic constraints, time scarcity, and the aggressive marketing of ultra-processed alternatives all operate simultaneously across individual, social, and environmental levels (4). Rounding out this section, Eşer Durmaz et al. show in a Turkish sample that women with pro-environmental attitudes toward food waste prevention also report greener dietary patterns, suggesting that environmental identity and dietary behavior are connected and may be co-leveraged in behavior change programming.

## 3 Food loss, waste, and the economics of sustainable food chains

Approximately one-third of all food produced for human consumption is lost or wasted globally, with associated environmental costs comparable to those of the world's third-largest emitter (5). Reducing food loss and waste is therefore an environmental, economic, and food security imperative. Two contributions in this volume approach that imperative from complementary angles: one quantifying the scale and environmental footprint of institutional waste, the other exploring the motivational dynamics of waste reduction among food professionals.

Abiad et al. quantify plate waste in a Lebanese hospital setting with striking precision, finding that nearly 31% of served food is discarded, with vegetables the most wasted food group and animal products accounting for the largest share of the resulting environmental footprint despite being served in smaller quantities. Their calculations of water, carbon, and nitrogen losses per hospital bed per day make the systemic scale of the problem concrete and actionable, pointing toward menu design, portion calibration, and patient preference profiling as priority targets. Zick et al. approach waste reduction through an entirely different lens, engaging professional chefs via Theory U, a change management methodology grounded in deep listening and collective reflection. Their study demonstrates that sustainable behavior adoption in culinary settings is not primarily a knowledge problem: chefs who engaged with Theory U developed stronger intrinsic motivation to reduce waste and greenhouse gas emissions and did so in ways authentic to their professional identities. The broader lesson extends across food system sectors: behavioral transformation is more durable when it emerges from within communities of practice rather than being prescribed from outside.

## 4 Governance, equity, and inclusive food systems policies

Even the most carefully designed agricultural innovations and dietary guidelines will fail to reach those who need them most if the governance systems through which they must travel are fragmented, underfunded, or captured by narrow interests. The seven contributions in this grouping examine the institutional, policy, and equity dimensions of food systems transformation across a wide range of contexts: Pacific Island governance structures, biofortification in Uganda, seed systems in Mali, food self-reliance in Puerto Rico, medically tailored meals in the United States, and infant feeding knowledge in the UAE.

Farmery et al. map multisectoral stakeholder visions for food systems governance across Pacific Island nations, finding that while articulated aspirations encompass nutrition, gender equity, and environmental stewardship, agricultural production priorities continue to dominate governance institutions in practice. This enduring sectoral dominance limits the transformative potential of reform even where stated goals extend well beyond productivity (6), and the paper makes a compelling case for deliberate institutional redesign rather than incremental adjustment. This argument carries particular weight in island contexts where fish and aquatic foods form the nutritional and cultural foundation of food systems yet remain underrepresented in the terrestrial-agriculture-dominated governance institutions that will shape their future. Alioma et al. examine governance bottlenecks to scaling iron- and zinc-biofortified crops in Uganda, identify coordination failures among research institutions, seed systems, and extension services as key constraints, and document how targeted training can meaningfully strengthen local actors' navigation of these systems. Their findings read naturally alongside Sylla et al.'s perspective from Mali, which shows how inclusive community seed systems that deliberately empower women as seed producers and custodians of varietal diversity can simultaneously expand access to nutritious crop varieties and strengthen local food system resilience.

Bezares et al. develop and apply a framework to estimate food availability and self-reliance in Puerto Rico, documenting deep structural vulnerability: the island imports the vast majority of its food, leaving the population exposed to price shocks, supply chain disruptions, and intensifying climate events that disproportionately affect small island territories. The Puerto Rico case is exemplary of a broader class of vulnerabilities facing small island developing states globally. Bezares et al. also make an implicit argument for the strategic value of decentralizing food production and shortening supply chains, a logic that runs equally through Sylla et al.'s findings on community seed systems in Mali. Food system resilience is built, in part, through local productive capacity and reduced exposure to supply chain fragility, a principle that food systems governance has been slower to operationalize than the evidence warrants. Folta et al. examine facilitators and barriers to enrollment in a medically tailored meals program within Massachusetts Medicaid, using clinic staff interviews to reveal a significant gap between policy design and implementation reality: staff knowledge deficits, time pressures, and patient-level barriers, including housing instability and cultural food preferences, all shape who ultimately receives the benefit. Equity-oriented nutrition programming demands sustained attention to implementation conditions, workforce development, and the lived experiences of both providers and recipients. Padayachee et al. close the Research Topic with a study of maternal knowledge, attitudes, and practices regarding camel milk as an infant food in the UAE, highlighting meaningful gaps in understanding of its protein composition and infant nutritional requirements. The contribution is a reminder that food system sustainability encompasses the knowledge systems shaping household food choices for the most nutritionally vulnerable family members, a dimension that formal policy processes frequently overlook.

## Conclusions

Twenty-three articles assembled in this Research Topic do not speak with a single voice; they come from different disciplines, different geographies, and different nodes of the food system. Yet read together, they converge on a coherent set of insights about what food systems transformation requires and where it most consistently breaks down. Three themes stand out.

First, transformation cannot be pursued along any single axis. Agricultural sustainability without nutritional adequacy is ecologically virtuous but nutritionally incomplete. A dietary transition without economic accessibility is aspirational but socially inequitable. Governance reform without active inclusion of women, smallholders, and marginalized communities' risks reproducing the very exclusions it seeks to correct. A related corollary is that food systems transformation focused mainly on terrestrial systems risks overlooking coastal, riverine, and island communities where aquatic foods are central, highlighting the need for a more inclusive perspective.

The triple convergence of economic, social, and environmental sustainability that frames this Research Topic is not a rhetorical device; it is a genuine analytical requirement. These dimensions must be addressed simultaneously and in relationship to one another, not sequentially by different disciplinary communities working in isolation.

Second, context is not a confounding variable; it is constitutive. The acceptability-sustainability tensions documented in New Zealand and the Netherlands differ fundamentally in character from the adoption barriers facing smallholder farmers in Malawi or the governance capture documented in Pacific Island nations. Sustainable food system solutions cannot be lifted wholesale from one setting and transplanted to another. They require deep local knowledge, participatory co-design with affected communities, culturally grounded implementation strategies, and governance structures genuinely responsive to local priorities. This is not an argument against generalizable science; it is an argument for science that takes contextual variation seriously as an object of inquiry rather than noise to be eliminated. A related gap is the limited attention to aquatic food systems and the coastal, island, and fishing communities that depend on them, highlighting the need to move beyond a predominantly terrestrial perspective.

Third, the persistent gap between scientific knowledge and policy practice remains the field's most urgent challenge. We know a great deal about what constitutes a healthy and sustainable diet. We know which agricultural practices protect soil health and farmer livelihoods. We know which governance designs support inclusive food systems, and which entrench narrow sectoral interests. What we have been less successful at is translating that knowledge into durable change at the speed and scale that converge planetary and public health crises demand. Accelerating that translation will require efficient institutions: multi-stakeholder platforms that genuinely integrate across sectoral silos, funding mechanisms that reward inter- and trans-disciplinary collaboration, mechanisms that incentivize sustainable practices across food value chains, and policy processes that routinely center on the perspectives of those most exposed to food system failures and most dependent on its transformation.

The guest editors are grateful to all contributing authors for their rigorous and illuminating work, to the reviewers who strengthened each contribution, and to the editorial teams at Frontiers in Nutrition and Frontiers in Sustainable Food Systems who made this volume possible. We hope that the researchers, policymakers, and practitioners who engage with these pages find in them both the intellectual grounding and the practical orientation to continue the difficult, essential, and ultimately hopeful work of building sustainable healthy food systems that nourish people and planet alike.
